# Atypical Antiglomerular Basement Membrane Disease in a Pediatric Patient Successfully Treated with Rituximab

**DOI:** 10.1155/2021/2586693

**Published:** 2021-07-17

**Authors:** Kuang-Yu Jen, Ari Auron

**Affiliations:** ^1^Department of Pathology and Laboratory Medicine, University of California, Davis School of Medicine, Sacramento, CA, USA; ^2^Department of Pediatric Nephrology, The Permanente Medical Group, Roseville, CA, USA

## Abstract

Classic antiglomerular basement membrane (anti-GBM) disease is an exceedingly rare but extremely aggressive form of glomerulonephritis, typically caused by autoantibodies directed against cryptic, conformational epitopes within the noncollagenous domain of the type IV collagen alpha-3 subunit. Pathologic diagnosis is established by the presence of strong, diffuse, linear staining for immunoglobulin on immunofluorescence microscopy. Recently, patients with atypical clinical and pathologic findings of anti-GBM disease have been described. These patients tend to have an indolent clinical course, without pulmonary involvement, and laboratory testing rarely reveals the presence of anti-GBM antibodies. Specific guidelines for the treatment and management of these patients are unclear. Here, we describe a case of atypical anti-GBM disease in a young child who presented with hematuria and prominent proteinuria. Throughout the course of his illness, creatinine remained normal. He was conservatively treated with steroids and rituximab, resulting in resolution of his clinical symptoms and normalization of laboratory findings.

## 1. Introduction

Antiglomerular basement membrane (anti-GBM) disease is an autoimmune disorder that is widely viewed as one of the most aggressive forms of glomerulonephritis. It can be accompanied by concurrent pulmonary hemorrhage, in which case the term Goodpasture syndrome is preferred. Anti-GBM disease is extremely rare in adults, with an estimated incidence of 0.5–1.64 cases per million per year, and even more so in children with under 30 cases ever reported in the literature [1–5]. A bimodal age distribution has been observed in larger series, showing peak incidences in the third decade and in the sixth to seventh decades [4, 6–8]. Those in the younger age group tend to present with Goodpasture syndrome and are more often males, whereas those in the older age group tend to experience disease limited to the kidneys.

Correlating to the rapidly progressive nature of this condition, crescents and necrotizing lesions are nearly always identified on biopsy, often showing diffuse glomerular involvement. Pathologic diagnosis necessitates the presence of diffuse, linear glomerular basement membrane staining for immunoglobulin (typically immunoglobulin G) on immunofluorescence microscopy, which is most commonly caused by the presence of autoantibodies directed against cryptic, conformational epitopes within the noncollagenous domain of the type IV collagen alpha-3 subunit (*α*3NC1) [9, 10].

More recently, it has become apparent that approximately 10% of patients with immunofluorescence findings of anti-GBM disease present with atypical clinical and pathological findings, often characterized by an indolent clinical course [11, 12]. Such patients show no pulmonary involvement, and laboratory testing rarely reveals anti-GBM antibodies against *α*3NC1. Nearly all cases thus far reported have been in adults, with very rare instances in the pediatric population [13, 14]. Here, we describe a case of atypical anti-GBM disease in a young child, who was conservatively treated with steroids and rituximab, resulting in resolution of clinical symptoms and normalization of laboratory findings.

## 2. Case Report/Case Presentation

A 4-year-old Caucasian boy with no significant past medical history developed nonspecific gastrointestinal and respiratory symptomatology, including vomiting, rhinorrhea, and cough that lasted for 4-5 weeks. Subsequently, he developed a fever followed by multiple episodes of painless gross hematuria that lasted for a few days. At that point, he was referred to pediatric nephrology for further workup. Initial physical examination was unremarkable with the exception of mild pallor. He was normotensive, afebrile, and did not have any rashes or joint pain. Pulmonary examination was normal. Urinalysis revealed the presence of blood with active urinary sediment on urine microscopy, as well as nephrotic range proteinuria of approximately 5.4 g/g based on a spot urine protein-to-creatinine ratio. Other laboratory findings were normal, including normal serum creatinine of 0.34 mg/dL. Computed tomography of the chest was normal. Renal ultrasonography revealed bilateral echogenic kidneys that were of normal size.

**Figure 1:**
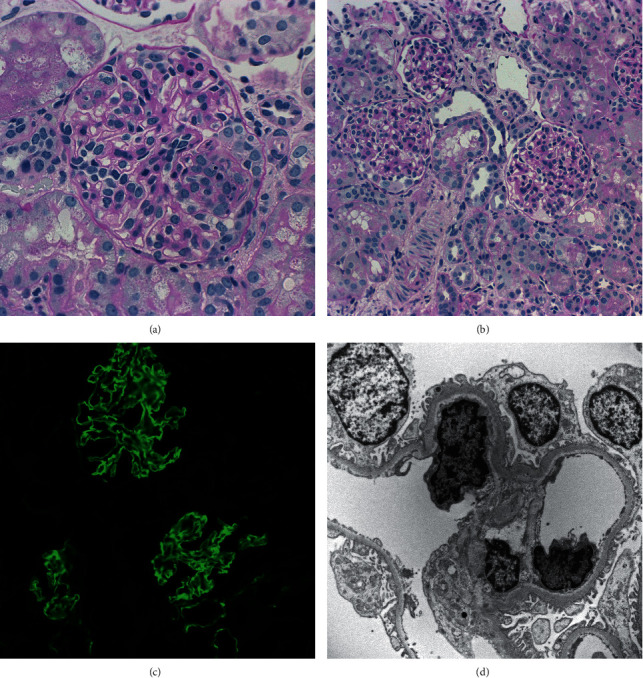
Renal biopsy findings. (a) One glomerulus showing segmental endocapillary hypercellularity, karyorrhectic nuclear debris, and a small cellular crescent (periodic acid-Schiff; original magnification: 400x). (b) Nearly, all of the remaining glomeruli demonstrate no significant abnormalities (periodic acid-Schiff; original magnification: 200x). (c) Immunofluorescence for IgG reveals diffuse, global, linear staining within the glomerular basement membrane (original magnification: 200x). (d) Widespread, subtle subendothelial widening is noted on electron microscopy, without the presence of any immune deposits (original magnification: 4800x).

Subsequently, a renal biopsy was performed. The light microscopy sections contained more than 36 glomeruli with 7 additional glomeruli on toluidine blue-stained thick sections, of which none were globally sclerotic. Two glomeruli showed subtle segmental scars, one of which was associated with karyorrhectic nuclear debris. Deeper level sections disclosed a single glomerulus with segmental endocapillary hypercellularity, karyorrhexis, and a small cellular crescent (Figure 1(a)). A few glomeruli exhibited prominent visceral epithelial cells with protein resorption droplets, while others demonstrated tubularization of the parietal epithelium. Aside from these glomerular changes, the remaining glomeruli appeared unremarkable (Figure 1(b)). The tubulointerstitium and vessels were also unremarkable, except for a few scattered red blood cell casts. Immunofluorescence revealed strong, diffuse, global, linear glomerular basement membrane staining for IgG as well as moderate kappa and lambda light chain staining in the same pattern (Figure 1(c)). Other immunoreactants including IgM, IgA, C3, C1q, fibrinogen, and albumin were negative. Electron microscopy disclosed widespread mild subendothelial widening, without the presence of electron-dense immune deposits (Figure 1(d)). Given the rare finding of active glomerulonephritis along with the strong linear IgG glomerular basement membrane staining on immunofluorescence, the diagnosis of atypical anti-GBM disease was made.

The biopsy findings prompted further laboratory studies, including serologies for anti-GBM antibody, which was positive at 6.3 AI (normal <1.0 AI) by multiplex flow immunoassay. All other serologies including antinuclear antibodies and antineutrophil cytoplasmic antibodies were negative. Complements C3 and C4 were within normal range.

The patient was treated with intravenous methylprednisolone (30 mg/kg) daily for 3 days followed by oral prednisone (2 mg/kg) daily for 6 weeks. Concurrently, he received 4 weekly doses of rituximab at 375 mg/m^2^ per dose, which were well-tolerated. This decision to use rituximab instead of plasmapheresis was made given the patient's normal serum creatinine and minimal pathology by light microscopy. His prednisone was then tapered off over the next 5 months. Once his oral steroid taper started, the patient was prescribed mycophenolate mofetil at 600 mg/m^2^ per dose, twice daily.

**Figure 2:**
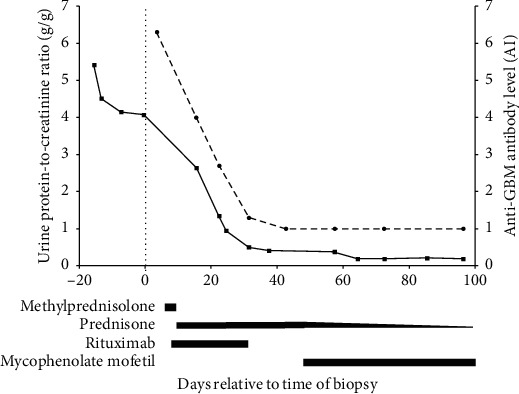
Degree of proteinuria and antiglomerular basement membrane (anti-GBM) antibody levels during the course of illness. Spot urine protein-to-creatinine ratio (solid line) and anti-GBM antibody levels (dotted line) are shown relative to the time of biopsy. ^*∗*^Limit of detection for anti-GBM antibody levels is 1.0 AI, which has remained <1.0 AI since day 43. ^*∗∗*^Limit of detection for urine protein-to-creatinine ratio is 0.19, which has remained between <0.19 and 0.25 since day 64. Data extend to day 334 at the latest follow-up. Length of treatment is indicated by black bars below the *X*-axis.

One week after receiving the fourth and final rituximab dose, the patient's anti-GBM antibody titers became undetectable and has stayed negative to date (Figure 2). Furthermore, one month after completing the rituximab course, the patient's proteinuria completely resolved and has remained negative to date (Figure 2). The patient's renal function throughout the course of his illness remained normal. Nine months after his initial presentation, the patient was asymptomatic, and at that point, slow tapering of mycophenolate mofetil was initiated and completely stopped 12 months after his initial presentation. He experienced no complications, such as infections or any significant side-effects, due to treatment. At the latest follow-up, 15 months after initial presentation, the patient remains asymptomatic, with normal urinalysis, urine sediment, blood pressure, and renal function, as well as negative anti-GBM titers.

## 3. Discussion/Conclusion

In the literature, the definition or inclusion criteria for atypical anti-GBM disease has been inconsistent. Numerous case reports use the term to describe patients who have biopsy-proven anti-GBM disease with the typical finding of crescentic/necrotizing glomerulonephritis but who are serologically negative for anti-GBM antibodies. Others use the term to describe anti-GBM disease in patients with secondary etiologies, such as infection or medication. Still others use the term to describe patients with primarily pulmonary symptoms, without significant renal involvement. In this report, we follow the inclusion criteria of Nasr et al. where atypical anti-GBM disease is defined by the presence of strong, diffuse, linear immunoglobulin staining within the glomerular basement membrane on immunofluorescence microscopy, but the light microscopy lacks the typical diffuse crescentic/necrotizing glomerulonephritis pattern of glomerular injury. Based on this definition, atypical anti-GBM disease is an extremely rare form of glomerulonephritis that affects approximately 10 percent of all patients that show the diagnostic finding of anti-GBM disease. In contrast to the rapidly progressive nature of classic anti-GBM disease, patients with this atypical variant present subacutely, with only mild renal insufficiency. The clinical course is usually indolent, although few may progress to end-stage renal disease. Thus far, nearly all patients that have been included in major case series reporting this entity have been adults. In this case report, we describe a case of atypical anti-GBM disease in a 4-year-old boy.

Cases of atypical anti-GBM disease in children are summarized in Table 1. It was first reported over 4 decades ago by Wilson and Dixon within a large cohort of almost all adult patients with classic anti-GBM disease [13]; however, specific clinical details were not provided. Other reported cases include an 8-year-old girl who was incidentally found to have anti-GBM disease based on screening urine studies that revealed nephrotic range proteinuria and hematuria. She was successfully treated with plasmapheresis, steroids, and cyclophosphamide [14]. To our knowledge, our patient is the first reported case of atypical anti-GBM disease in a child where instead of plasmapheresis, a more conservative treatment approach was taken with the use of rituximab.

Much of what is known about the clinicopathologic characteristics of atypical anti-GBM disease comes from two recently published major case series [11, 12]. The largest was reported by Nasr et al. on 20 patients, all of whom were adults [11]. Their patients often presented with heavy proteinuria as well as hematuria and mild renal insufficiency. None of them showed pulmonary involvement, and no circulating anti-*α*3NC1 antibodies were detected by conventional enzyme-linked immunosorbent assay (ELISA) or multiplex flow immunoassay in any of the patients. One-year patient and renal survival rates were 93% and 85%, respectively, indicating an indolent but sometimes slowly progressive clinical course. Interestingly, they noted that half of their patients showed monotypic immunoglobulin deposition, while the other half was polytypic. They reported no significant clinical or pathologic differences between the two groups except for a lower rate of crescents and/or fibrinoid necrosis in the monotypic cases.

The other major case series, by Troxell and Houghton, described 5 adult cases of atypical anti-GBM disease [12]. All four patients with urinalysis data presented with hematuria, and 2 of 3 patients with data on proteinuria status showed >1 g/g urine protein-to-creatinine ratio, although they excluded patients with heavy proteinuria from their series. In contrast to Nasr et al., 3 of their patients showed positive anti-GBM antibody serologies, of which one reported hemoptysis. This latter patient had a more unusual clinical course given that he initially exhibited features of Goodpasture syndrome with minimal renal involvement but 3 months later presented with more typical/classic features of anti-GBM disease, suggesting that an atypical presentation may indicate a prodrome to outright expression of classic anti-GBM disease.

Our patient's clinical presentation was similar to most of the reported cases of atypical anti-GBM disease, mainly consisting of proteinuria (nephrotic range by urine protein-to-creatinine ratio) and hematuria (both macroscopic and microscopic). However, in our case, the renal function on presentation and throughout the observation period remained normal. His biopsy revealed focal/rare endocapillary proliferative glomerulonephritis with small crescent formation involving <5% of the glomeruli, a pattern that was most frequently encountered in the case series summarized above. Interestingly, electron microscopy in our case exhibited widespread mild subendothelial widening, a common but nonspecific finding that can be seen in the setting of classic anti-GBM disease. Immunofluorescence microscopy revealed the presence of polytypic anti-GBM IgG given that both kappa and lambda light chains were positive. In contrast to the patients in the series by Nasr et al., our patient had detectable anti-GBM antibodies, which rapidly decreased to undetectable levels following treatment. Given that the anti-GBM antibodies in our patient were detected via multiplex flow immunoassay, these antibodies were most likely directed against the most common epitope typically seen in classic anti-GBM disease, namely, the type IV collagen *α*3NC1 domain. It is unclear why our patient experienced relatively mild renal symptoms even with appreciably high initial levels of anti-GBM antibodies. Possibilities include low epitope affinity by our patient's autoantibodies or a low degree of complement activation. To the latter point, it should be noted that C3 and C1q were negative by immunofluorescence microscopy on the biopsy, and thus, a low degree of complement activation could be plausible. In the case of low epitope affinity, the ability to detect the anti-GBM antibody by multiplex flow immunoassay would also be expected to be hampered and thus would be unlikely.

In classic anti-GBM disease, initial aggressive therapy is warranted, consisting of plasma exchange, oral cyclophosphamide, and glucocorticoids for all patients with kidney involvement who do not require immediate dialysis [9, 15]. However, the proper treatment modality for atypical anti-GBM disease is uncertain given the rarity of this disease. One must weigh the possible benefits of prompt and aggressive treatment to prevent any further renal injury against the detrimental cytotoxic effects of cyclophosphamide, especially for a child in our case. Given the patient's stable and baseline serum creatinine as well as the relatively mild histologic changes seen on biopsy, we decided to conservatively manage our patient with an initial combination of rituximab and steroids followed by mycophenolate, without the use of plasmapheresis/plasma exchange or cytotoxic agents. The patient immediately responded with a dramatic decrease in proteinuria and anti-GBM antibody levels, both of which have remained undetectable at the latest follow-up approximately 1-year after initial presentation. Given the success that we observed with conservative treatment of atypical anti-GBM disease in our patient, such an approach in children should be considered if this rare entity is encountered.

## Figures and Tables

**Table 1:** Pediatric cases of atypical antiglomerular basement membrane disease in the literature.

Publication	Age/gender	Presentation	Biopsy findings	*α*3NC1 antibodies	Treatment	Outcome
Jen and Auron (present case)	4/M	Nephrotic-range proteinuria, hematuria, normal renal function	One glomerulus with segmental endocapillary hypercellularity, karyorrhexis, and small cellular crescent	Positive	Rituximab, steroids, MMF	Asymptomatic with normal renal function at 15 months

Wilson and Dixon [13]	14/M	No onset of renal failure within a large cohort of typical anti-GBM disease	N/A	Positive	Steroids	Living 1 yr with normal function
	13/M			Negative	Steroids, azathioprine	Living 18 mo with normal function
	5/M			Negative	Steroids, cyclophosphamide	Living 7 yr with normal function

Nagano et al. [14]	8/F	Incidental hematuria and proteinuria on screening	Mild mesangial proliferation; no crescents	Positive	Plasmapheresis, steroids, cyclophosphamide	Decreased proteinuria and antibody titers; no long-term follow-up

## Data Availability

Data are available from the authors upon request.
